# Investigation of Morphological Changes of the Soft Tissue in the Aesthetic Zone: A 3D Virtual Analysis after Conventional Tooth Extraction and Benex^®^ Extraction

**DOI:** 10.3390/dj12080252

**Published:** 2024-08-09

**Authors:** Mayte Buchbender, Lotta Gath, Fabian Jaeckel, Anna Seidel, Marco Rainer Kesting, Manfred Wichmann, Werner Adler, Ragai Edward Matta

**Affiliations:** 1 Department of Oral and Maxillofacial Surgery, Friedrich-Alexander-Universität Erlangen-Nürnberg, 91054 Erlangen, Germany; lotta.gath@uk-erlangen.de (L.G.); marco.kesting@uk-erlangen.de (M.R.K.); 2 Department of Prosthodontics, University Hospital Erlangen of Friedrich-Alexander Universität Erlangen-Nürnberg, Glueckstrasse 11, 91054 Erlangen, Germany; fabian.jaeckel@hotmail.de (F.J.); anna.seidel@uk-erlangen.de (A.S.); manfred.wichmann@uk-erlangen.de (M.W.); ragai.matta@uk-erlangen.de (R.E.M.); 3 Department of Medical Informatics, Biometry and Epidemiology, University of Erlangen Nuremberg, 91054 Erlangen, Germany; werner.adler@uk-erlangen.de

**Keywords:** Benex^®^ system, less traumatic extraction, volume changes after extraction, intraoral scan

## Abstract

Background: Postoperative resorption of hard and soft tissues occurs as a consequence of tooth extraction. The Benex^®^-Control extractor allows minimally invasive extraction of the tooth without causing pronounced iatrogenic trauma. The aim of this study was to verify whether the resorption of the tissues after extraction can be reduced by using the Benex^®^ system compared to the conventional extraction method. Methods: Postoperative intraoral scans were superimposed after surgery (t0), after 7 days (t1), after 14 days (t2), after 30 days (t3), after 60 days (t4), and after 90 days (t5) within the two groups (study n = 14, control n = 16), and defined regions of interest (ROIs) (1–8) and volume changes were analyzed. In addition, the influence of gingival thickness and the thickness of the labial cortical plate was investigated. Results: The greatest decrease in volume was observed in both groups in ROI3, although there was no significant difference observed between the groups. In the presence of an adjacent tooth, there was less volume loss in the affected ROIs (1, 2 and 7, 8). The thickness of the gingiva and the bony lamella did not significantly influence the change in volume. Conclusions: Due to the small cohort, the results are limited, and the hypothesis is rejected.

## 1. Introduction

The aim of gentle extraction, especially in the anterior region, consistently involves the preservation of as much bone as possible, especially the labial cortical plate and, thereby, the soft tissue (the gingiva). The concept of immediate implant placement is ultimately based on this scenario. However, even if immediate implant placement is not performed, the extraction should be as gentle as possible to avoid major resorption processes of hard and soft tissues to achieve a maximum long-term aesthetic outcome. Both tissues must be measured in different ways, and various factors play a role in these effects, such as the thickness of the facial bone and the gingiva or the location of the tooth [1]. Experimental studies have shown that the bundle bone (as a tooth-dependent structure) resorbs after tooth extraction, thus resulting in a vertical bone loss of up to 2.2 mm on the facial side, which is less severe in the lingual aspect. This is based on the reduced thickness of the facial bone [2]. In clinical studies, a resorption of the alveolar ridge of up to 50% is observed in the first year after extraction, with two-thirds of this resorption occurring in the first 3 months [3]. Decreases of 2.6–4.5 mm in width and 0.4–3.9 mm in height were demonstrated by a systematic review [4]. In the anterior maxilla, 90% of the facial bone is less than 1 mm, and even less than 0.5 mm represents less than 50% of the bone; moreover, these values can be measured intraoperatively by using a periodontal probe or cone beam computed tomography (CBCT) [5,6,7,8]. At a thickness of 1 mm or less, a vertical bone loss of 62% of the original alveolar ridge can be observed. With a facial bone thickness greater than 1 mm, only 9% vertical bone loss occurs after 8 weeks [9]. This decrease was observed with healthy adjacent teeth and single extractions, particularly in the central part of the socket.

Due to the composition of thick, soft tissues (with more extracellular matrix and collagen), they are more favorable for healing in terms of surgical interventions [10,11,12,13]. The thickness of the facial soft tissue in the anterior maxilla in humans ranges from 0.5 to 1 mm, regardless of the thickness of the underlying facial bone [14,15,16]. In post-extraction sockets with thick facial bone, unchanged soft tissue is observed [14]. However, in contrast to the assumption of a decrease in soft tissue in thin bone types, an increase in soft tissue is observed, particularly in the crestal area [17,18,19]. It is assumed that faster resorption of the bone favors an increase in soft tissue.

Therefore, a number of clinical studies have already been performed to research the gentlest extraction method. This approach should also be efficient and involve few complications. Traditional forceps and elevators often result in the loss of the buccal bony plates [20,21]. In a systematic review, 11 articles with conventional extraction methods (forceps) and physical forceps were included, from which a total of 1028 teeth were extracted. The physical forceps methodology performed significantly better in terms of operation time. The same effect was observed for pain; however, the difference was not significant. Nevertheless, the authors concluded that physical forceps were more suitable for premolar and molar extractions [22]. In a retrospective study, an electromagnetic device was compared with conventional tooth extraction in 48 patients with regard to the digital volume changes of the socket [23]. The digital impressions that were obtained by using an intraoral scanner were compared with the initial scans after extraction and after 4 months. The volume loss in the electromagnetic extraction group was not significantly lower than that in the conventional extraction group. The final alveolar ridge volume after 4 months was 0.87 ± 0.34 cm^3^ in the study group and 0.66 ± 0.19 cm^3^ in the conventional extraction group.

A vertical extraction system was previously used in a clinical study as a proof of principle [24]. The authors concluded that the Benex^®^ system represents an atraumatic extraction method, particularly for single-rooted teeth. However, as there are only a few prospective studies comparing this system with conventional tooth extraction, especially in the maxillary anterior region, this study was designed with the hypothesis that there is less volume loss after tooth extraction with the Benex^®^ system than after conventional extraction by using forceps and elevators.

## 2. Materials and Methods

### 2.1. Study Design and Patient Selection

**Figure 1:**
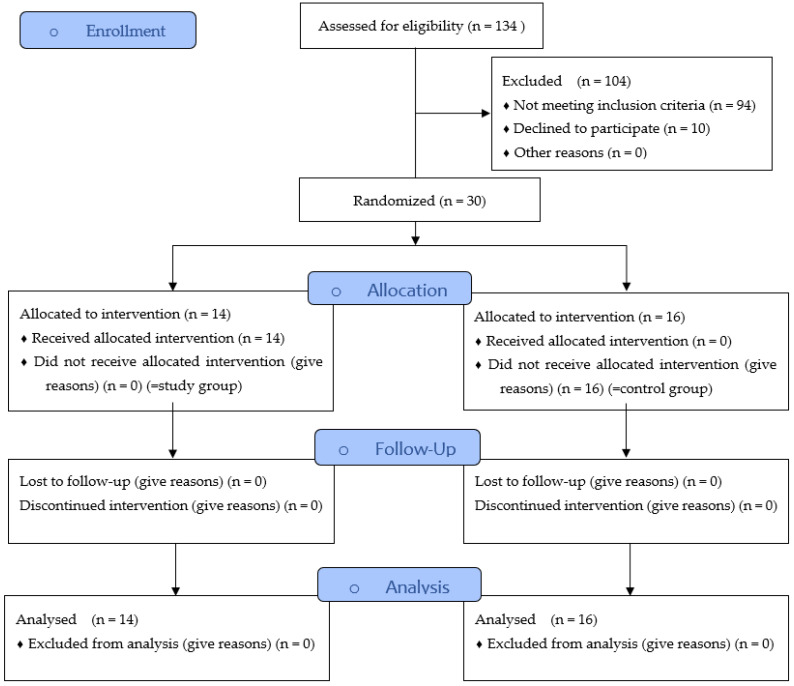
Study Flow Diagram.

In the present prospective randomized clinical trial (Figure 1), patients from August 2020 to January 2022 were enrolled and examined for morphological changes in hard and soft tissues following tooth extractions. The test subjects were then randomly assigned to two groups. The control group was subjected to conventional extraction (by forceps and levers). The Benex^®^ group, in which the extraction was performed by using a Benex^®^-Control vertical extractor (Hager & Meisinger, Neuss, Germany), was indicated as the study group. Only root residues without carious destruction were included and randomly assigned to the groups.

To obtain information on the morphological and volumetric changes during the healing process, digital impressions were taken with an intraoral scanner (Trios 4, 3Shape, Copenhagen, Denmark) at several time points (before and immediately after surgery [t0], after 7 days [t1], after 14 days [t2], after 30 days [t3], after 60 days [t4] and after 90 days [t5]). To determine the changes, the impressions were superimposed on each other for mutual comparison by using CAD analysis software (GOM Inspect, GOM GmbH, Braunschweig, Germany).

The subjects were included according to the criteria and visited either the Department of Oral and Maxillofacial Surgery, University of Erlangen-Nürnberg, or the Department of Prosthodontics, University of Erlangen-Nürnberg, in need of surgical tooth extraction. Ethical approval (No. 42_20 B; 25 May 2020) for this study was obtained from the ethics committee of the medical faculty of Friedrich-Alexander University Erlangen-Nuremberg, Germany, and the guidelines of the Declaration of Helsinki were followed. The study was also registered in the German clinical trial registry (DRKS00024089). Adult patients of all ages and sexes provided informed consent before they were included in the study. Furthermore, each patient was free to terminate participation in the study at any time without providing reasons. If one of the potential test subjects did not agree with the performance of the study or did not wish to participate in the study, they did not suffer any disadvantage in further treatment as a result of their decision.

The inclusion criteria for both groups were as follows:

- At least one tooth of the maxillary anterior region (regions 13 to 23), which was classified as not worth preserving on the basis of the extraction indications (to verify the suitability for extraction beyond any doubt, a radiological diagnosis of the respective tooth was routinely performed);
- Smokers were divided into two groups based on their consumption (moderate: <10 cigarettes per day and heavy: >10 cigarettes per day).
- The exclusion criteria for both groups were as follows:
- Inflammation of the periodontium or gingiva (periodontitis or gingivitis) in the extraction area;
- Previous mucogingival surgery, such as root tip resection;
- Pregnant or breastfeeding patients;
- Current or previous radiotherapy of the head and neck area or chemotherapy;
- Systemic medication could affect the outcome of the therapy. These included medications that can induce gingival hyperplasia (anticonvulsants, immunosuppressants, and calcium antagonists), as well as antiresorptive agents (bisphosphonates and monoclonal antibodies).

### 2.2. Surgical Procedure

**Figure 2:**
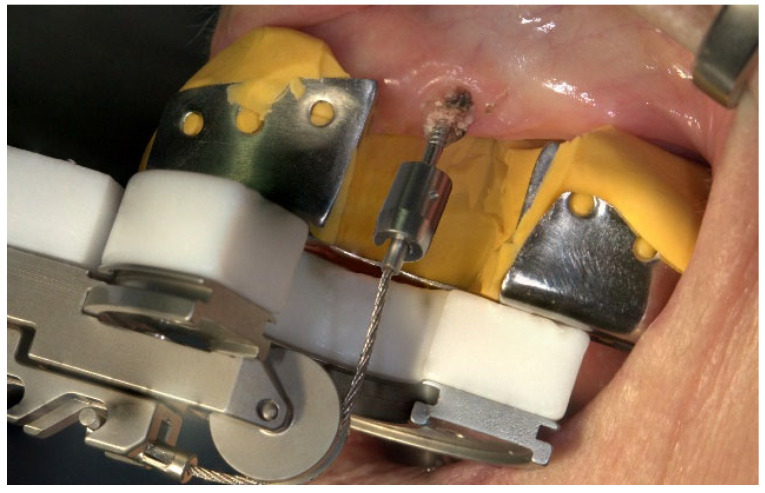
Mode of operation of the Benex^®^ extraction system on the upper jaw in the anterior region.

All of the surgical procedures were performed under local anesthesia (Ultracain^®^ DS; adrenaline 1:200,000; Sanofi-Aventis GmbH, Frankfurt, Germany). Surgical extraction in both groups was performed by one experienced oral surgeon at the Department of Oral and Maxillofacial Surgery who had already used the Benex^®^ system for at least 2 years prior to this study. In the test group, the teeth were extracted as gently as possible for routine tooth extraction treatment with common instruments, such as forceps. In the study group, the teeth were extracted according to the Benex^®^ system (Figure 2). After extraction, the wound edges were routinely adapted by cross-stitching with Vicryl^®^ 5-0 Rapide (Ethicon GmbH & Co KG, Norderstedt, Germany). In individual cases, the extraction was radiologically verified. At the end of each intervention, the patients were instructed in terms of nutrition and postoperative events, such as bleeding or pain and swelling; moreover, they were administered painkillers if needed. Moreover, follow-up appointments were scheduled.

### 2.3. Study Parameters

The following clinical parameters were collected at each visit and time point of the study:

- The biotype of the gingiva was measured with a periodontal probe (PCP 12, Hu-Friedy, Frankfurt am Main, Germany) and classified as thin, mixed, or thick.
- The following intraoperative clinical parameters were utilized and tactile measured by the surgeon with a periodontal probe:
  - Presence of the labial cortical plate (0: no, 1: yes, 2: fenestrated) [25];
  - Thickness of the labial cortical plate (in the orofacial direction, 0: not present, 1: ≤1 mm, 2: >1 mm) [9];
  - Vestibular thickness of the gingiva (0: <1 mm, 1: 1–2 mm, 2: >2 mm) [26].

#### Outcomes

The primary outcome was defined as the volumetric change after extraction between the study group and control group. The secondary outcome was defined as the difference between the groups in terms of the following:

- Influence of the gingival biotype on the volume change;
- Influence of the thickness and texture of the labial cortical plate on the volume change;
- Influence of the presence or absence of the mesial or distal adjacent tooth on the volume change.

### 2.4. Digital Examination

**Figure 3:**
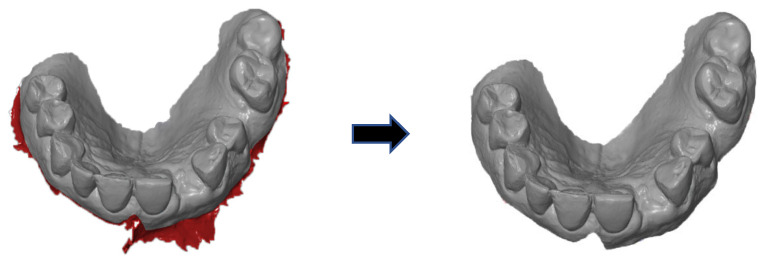
Editing of the scans in terms of trimming the contours.

**Figure 4:**
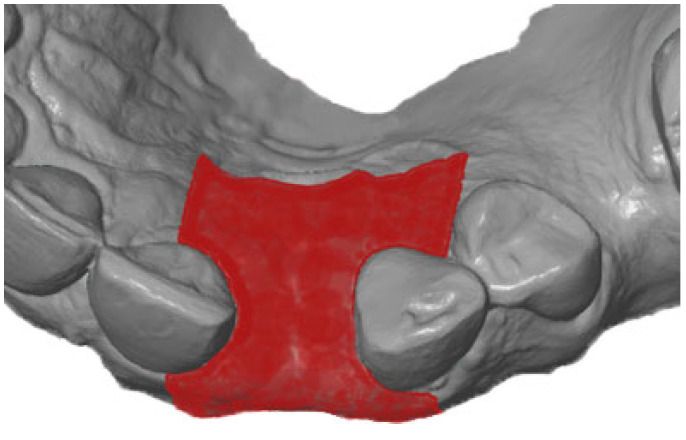
Defining the region of interest after extraction of the teeth.

**Figure 5:**
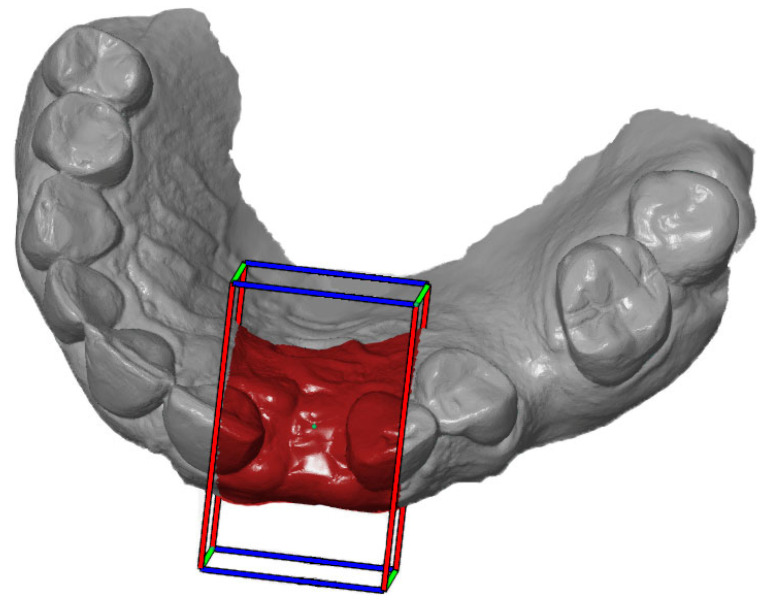
Definition of the ROI according to the cuboid.

**Figure 6:**
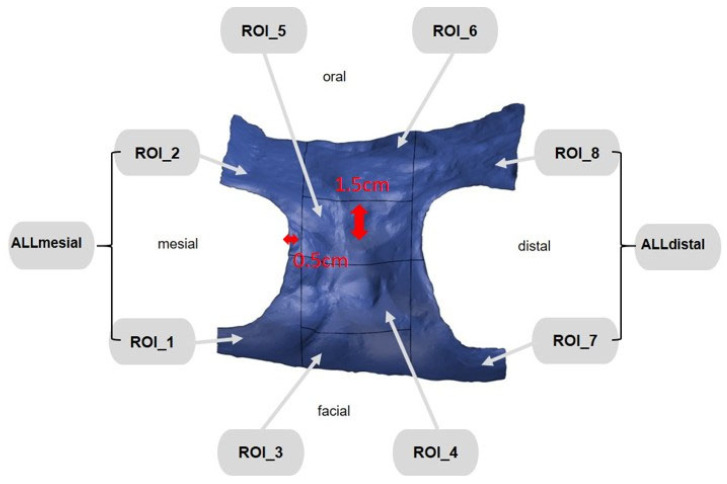
ROI subdivided into 10 different areas.

**Figure 7:**
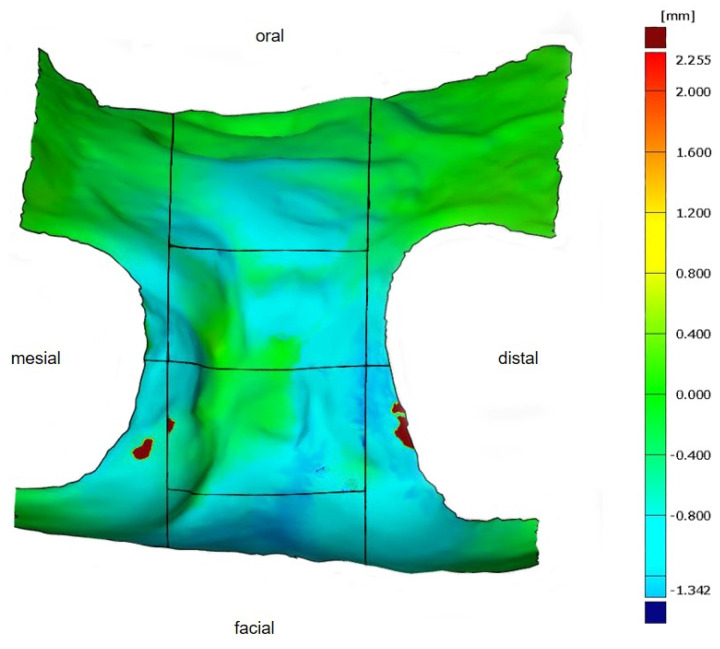
Determined volumetric change in the defined ROI in a color-coded distance map for better visualization. As seen from the scale (right), volume increases are shown in red, no changes in green, and volume decreases in blue.

First, the postoperative scan (t0), which served as a reference for all of the further scans of the follow-up controls, was imported into the program and edited (Figure 3). To make the analysis comparable between different subjects, the three-dimensional scans of the maxilla were limited to a specific region of interest (ROI) (Figure 4). For this purpose, a cuboid with defined side lengths was used depending on the extracted tooth (Table 1 and Figure 5). To interpret separate areas of the entire ROI, it was segmented into 10 different subareas (Figure 6). The subdivision was performed on the basis of the anatomical conditions and defined as follows. At a distance of 0.5 cm from the adjacent tooth, the area from facial to oral was defined as the ALLmesial and ALLdistal. The further subdivision was based on the center of the socket and that of the adjacent teeth, whereby the entire ROI and the described areas (ALLmesial and ALLdistal) were again divided centrally (Figure 6). Finally, the two resulting central areas were subdivided at the edge of the socket in facial and oral terms at a distance of 1.5 cm. This ROI was used to match all of the subsequent scans to define the changes in volume in terms of an area comparison (Figure 6). The volumetric changes in these 10 areas during the five follow-ups (t1, t2, t3, t4, and t5) were compared to those of the first postoperative scan (t0). The STL files to be superimposed comprised of entirely closed geometries. In order to calculate a change in volume, the models were superimposed. The matching was refined using the “local best fit” function of the program. The tooth surfaces of the immediate neighboring teeth were selected mesial and distal as congruent areas in the ACTUAL model (scan at t0). This step made it possible to avoid inaccuracies in the superimposition, particularly in the area of the ROI. As a result, the digital maxillary model of the first control appointment was aligned congruently and automatically with the TARGET (scans at t1-t5) model. The analysis methodology was used to calculate the discrepancy between the TARGET and ACTUAL models (area comparison). This involved calculating the valid surface area in square millimeters (mm^2^, Table 1) and the average deviation of all measurement points between the target and actual models in millimeters (mm) within the regions of interest (ROIs). The distances between corresponding mesh vertices (closest point) on the superimposed surfaces were then displayed in a color-coded distance map (Figure 7). By multiplying the corresponding surface area by the deviation of each point measured within the ROI, the total volume in cubic millimeters (mm^3^) was calculated by the software as the sum of all such calculations. It represents the average volume increase or decrease in three dimensions in the space between the two surfaces being compared.

This was performed in order to display the remodeling of hard and soft tissue in all three dimensions. However, since the ROIs varied in size between individuals (differing valid surface area), the ratio between the total volume and area was calculated (total volume [mm^3^]/valid surface area [mm^2^]; Table 2) to accurately display the actual change in volume.

#### Statistical Analysis

The collected data were statistically analyzed by using R software, version V4.2.0. (R Core Team (2018). R: A language and environment for statistical computing. R Foundation for Statistical Computing, Vienna, Austria). The influence of the various factors on the change in volume was investigated by using mixed linear models. The quotient of the total volume to the surface area (Vol/Area) was used as the dependent variable. Different variables were defined as being independent for different questions:

1. Timepoints t1 and t5 and groups (study, control), ROIs (3, 4, 5, ALLmesial, Alldistal);
2. Timepoints t1 and t5 and groups (study, control);
3. Groups (study, control), ROIs (1, 3, ALLmesial), adjacent tooth mesial (ATmesial);
4. Groups (study, control), ROIs (3, 7, ALLdistal), adjacent tooth distal (ATdistal).

The Mann–Whitney U test or analysis of variance (ANOVA) was used to compare unrelated groups with a significance level of *p* < 0.05.

The results are visualized in boxplot diagrams and descriptive tables.

## 3. Results

### 3.1. Study Cohort

In total, thirty subjects (control group, n = 16; study group, n = 14) were included in this study. There were no registered complications in either group, and the extraction sockets were completely healed regardless of the study parameters. Detailed information about sex, age, location of the teeth, and status of nicotine abuse in both groups is listed in Table 3. Based on the total operation time, a mean treatment time of 73.9 ± 13.5 min was determined for the conventional extraction method and 71.5 ± 23.3 min for the Benex^®^ method. The statistical comparison showed no significant difference (*p* = 0.221); however, the average operation times of both groups were similar to each other.

### 3.2. Primary Outcome

The detailed values for the control group are shown in Table 4. A decrease in volume was recorded in ROI3 at time t1 in the compared time periods, whereas an increase occurred in the other ROIs (4, 5, ALLmesial, ALLdistal) at the same time. At timepoint t5, the largest decrease in volume was observed in ROI3, although the other ROIs (4, 5, ALLmesial, ALLdistal) also showed a decrease. The differences were not statistically significant.

**Figure 8:**
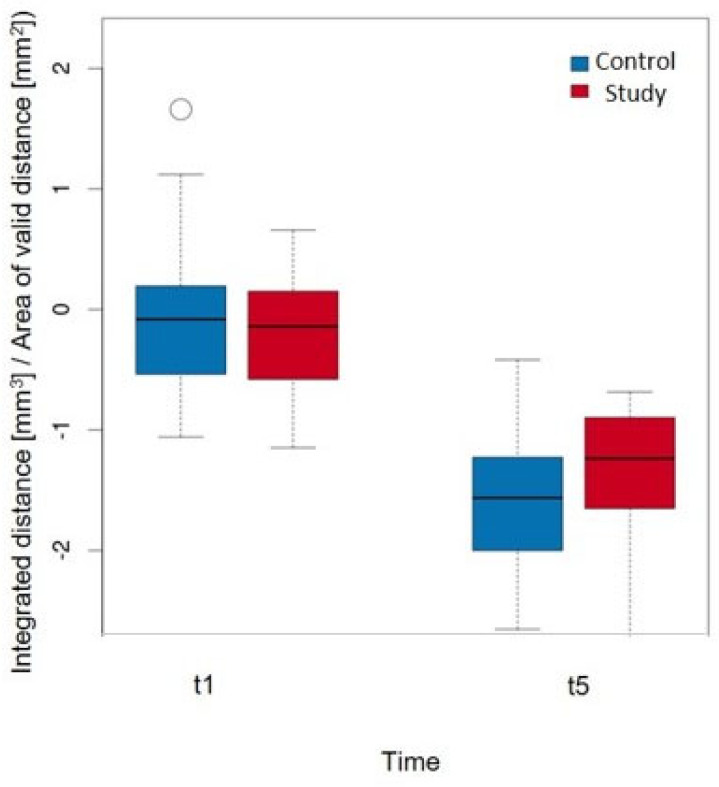
Comparison of the volume change per area (mm^3^/mm^2^) in ROI_3 at timepoints t1 and t5 when considering the groups with a *p*-value = 0.737.

The detailed values of the study group are shown in Table 5. At timepoint t1, the greatest decrease in volume was observed in ROI3, whereas the ROIs (4, 5, ALLmesial) showed an increase in volume. ROI3 also showed the greatest decrease in volume at time t5, with all of the other ROIs (4, 5, ALLmesial, ALLdistal) also showing a decrease. The linear model (Table 6) clearly showed that the volume decrease over the entire observation period was significantly greatest in ROI3. However, this was not statistically significant in terms of group affiliation; herein, it is only possible to derive a tendency for the study group (it experiences a smaller volume decrease). This trend (without statistical significance) can also be observed in the isolated comparison of ROI3 between t1 and t5 (Figure 8). The decrease in volume was greater in the control group at t5 than in the study group.

### 3.3. Secondary Outcomes

#### 3.3.1. Influence of the Gingival Biotype on the Volume Change

In the control group, patients with a thick biotype exhibited the greatest volume loss (−1.858 ± 0.753 mm^3^/mm^2^), and patients with a thin biotype exhibited the least volume loss (−1.277 ± 0.417 mm^3^/mm^2^). The values for the mixed biotype were observed between these values (−1.551 ± 0.645 mm^3^/mm^2^).

A similar distribution was observed for the study group. The greatest volume loss was detected in the thick biotypes (−1.522 ± 0.766 mm^3^/mm^2^), and the smallest volume loss was detected in the thin biotypes (−0.985 ± 0.228 mm^3^/mm^2^). The values for the mixed biotypes (−1.340 ± 0.396 mm^3^/mm^2^) were observed in the middle of the two maxima.

However, in summary, no significant difference could be detected with regard to the volume change, neither for the biotypes (*p* = 0.142) nor for the different groups (*p* = 0.159).

#### 3.3.2. Influence of the Thickness and Texture of the Labial Cortical Plate on the Volume Change

The distribution of the mean values in the control group demonstrated the greatest volume change at a thickness ≤1 mm (−1.911 ± 0.610 mm^3^/mm^2^). The smallest change occurred when the labial cortical plate was not present (−1.348 ± 0.656 mm^3^/mm^2^), and there was a value observed for a thickness of >1 mm (−1.390 ± 0.512 mm^3^/mm^2^).

In comparison, the study group showed a different distribution. The greatest change occurred at an average thickness >1 mm (−1.691 ± 0.959 mm^3^/mm^2^), and the lowest change occurred at an average thickness ≤1 mm (−1.161 ± 0.349 mm^3^/mm^2^). In the middle of the maxima, the mean value of the change in subjects who had no labial cortical plate was observed (−1.540 ± 0.183 mm^3^/mm^2^).

This aspect of the statistical analysis did not detect any significant influence of the thickness of the labial cortical plate (*p* = 0.562) or group (*p* = 0.203) on the volume change per area (mm^3^/mm^2^).

The volume change within the control group with a nonintact labial cortical plate was −1.544 ± 0.682 mm^3^/mm^2^, and that with an intact labial cortical plate was −1.669 ± 0.651 mm^3^/mm^2^. The mean values of the study group were also similar to each other: nonintact labial cortical plate (−1.395 ± 0.361 mm^3^/mm^2^) and intact labial cortical plate (−1.396 ± 0.678 mm^3^/mm^2^). Therefore, neither a significant result with regard to the condition of the labial cortical plate (*p* = 0.721) nor the group affiliation (*p* = 0.279) could be calculated.

#### 3.3.3. Influence of the Presence or Absence of the Mesial or Distal Adjacent Tooth on the Volume Change

Table 7 shows that patients with an adjacent mesial tooth had significantly less volume loss at time t5 compared to those without (*p* = 0.001). Specifically, ROI1 (0.592 mm^3^/mm^2^) and ROI_ALLmesial (0.993 mm^3^/mm^2^) had significantly less volume loss than ROI3 (*p* < 0.001). Conversely, although not statistically significant (*p* = 0.060), there was a trend towards lower volume loss in the study group with an adjacent mesial tooth across all ROIs.

Table 8 demonstrates that patients with a distal adjacent tooth had a significantly higher value of 0.300 mm^3^/mm^2^ (*p* = 0.037). ROI7 (0.374 mm^3^/mm^2^) and ROI_ALLdistal (0.835 mm^3^/mm^2^) experienced significantly less volume loss compared to ROI3 (*p* < 0.001). Similarly, there was a nonsignificant trend towards lower volume loss in the study group with a distal adjacent tooth across all ROIs (*p* = 0.174).

## 4. Discussion

Currently, there are a variety of surgical techniques for implantation or hard and soft tissue augmentation from which surgeons can choose for their patients. However, with the ever-increasing number of PROM-associated publications (patient-reported outcomes) [27], some procedures, such as complex bone augmentations, are becoming unsuitable. Therefore, it is important to perform the procedure as gently as possible, one step beforehand, such as during tooth extraction, to maximize the preservation of hard and soft tissues. Tooth extraction has consistently represented trauma to these tissues but can be limited by certain extraction methods [28]. Vertical extraction systems such as the Benex^®^ system are most commonly used for this purpose.

To the best of our knowledge, this system was first examined in 72 patients in 2013 [24]. The Benex^®^ system was used in 111 tooth extractions due to caries or root residues in which the usual forceps were inappropriate for use. Of these, 92 teeth were successfully extracted, with a higher success rate for single-rooted teeth (89%). The majority of failures were associated with insufficient screw retention, root fractures, or incorrect insertion of the screw and associated root fractures during extraction by using vertical traction. The time for the operation was also noted in this study and improved over the course of the study, with an average of 10.5 min per procedure. Ultimately, the procedure time was less than 2 min in 64% and less than 4 min in 80% of the patients.

The selection of indications in the study showed that the primary aim at the time was not to protect hard and soft tissues but to identify an option in which the previous methods functioned less optimally. However, the investigation of resorption and volume loss was addressed by these authors at the conclusion of further studies. In the 19 patients for whom Benex^®^ was not used, flap surgery was performed in 8 patients. In these patients, between 2 mm and more than 4 mm of bone had to be removed. However, in our study, the investigation of the different extraction methods focused on losing as little volume of hard and soft tissues as possible; although not significant, this effect could be shown with a tendency toward the Benex^®^ system, especially in the analyzed vulnerable facial aspect of the socket, which had the greatest decrease in volume after a 90-day follow-up. At that time period of 90 days, the other analyzed ROIs of the socket also showed a decrease in volume, which is comparable to what is known in the literature after extraction.

In addition, only single-rooted teeth were extracted in the highly aesthetic anterior region, wherein volume preservation is particularly important with regard to possible later implant placements for restoration. In the group without Benex^®^, flap surgery was not performed at any time for removal but was gently performed with appropriate periotomes and forceps, which may also be a reason why the difference in volume changes between the groups was not significant. The literature has demonstrated that even the formation of a flap without further bone removal leads to increased bone resorption from the bony surface [29]. In particular, in the first 4–8 weeks, flapless tooth extraction results in less bone resorption than does full-thickness flap extraction [30]. In terms of operation time, the two extraction methods that were investigated in our study showed no significant difference and corresponded to those of the previous literature. However, no PROMs were examined in our study, although it can likely be assumed that patients consider the Benex^®^ appliance to be less comfortable than it is for conventional extraction.

Another prospective observational proof of principle clinical study compared Benex^®^ vs. conventional extraction and, in the event of failure, flap surgery with and without bone removal [31]. A total of 276 out of 323 teeth in 240 patients were successfully removed with the Benex^®^ system. The authors hypothesized that fewer flap surgeries would be required when using the Benex^®^ system. Of the 47 failures, 18 flap surgeries were necessary for tooth removal. Failures were increased in multirooted teeth, root canal-treated teeth, and maxillary lateral incisors. In the conventional extraction group, a total of 1719 teeth were removed, 21 of which required flap surgery. Thus, a high number of teeth proves that the Benex^®^ system works well. Ultimately, the question of why flap surgery should be performed in both groups in the event of failure remains unanswered. The reason probably lies in the fact that 69.7% of multirooted teeth were included. In the case of failure of the Benex^®^ system in our study, the teeth were excluded from the study and did not involve any of the patients in either group. However, we did not have to perform flap surgery or the use of rotary instruments to remove those, although this was not to be expected due to the preselection of anterior teeth in the maxilla. Our observations were also not consistent with regard to the lateral incisors, which could fail more frequently on more delicate root structures, as discussed in another study.

In a more recent randomized clinical study, parameters for the use of the Benex^®^ system were also used as the methodology, and the success of the extractor could be proven on the basis of many teeth [32]. These researchers investigated the postoperative parameters of socket wound healing and complications or pain that was experienced after Benex^®^ in comparison to conventional extraction. The aim of this study was to improve patient quality of life through these outcomes. Thirty-eight patients with single-rooted non-restorable teeth were included and randomly divided equally into two groups. To evaluate the complications and pain experience, a questionnaire with a pain numeric scale was used, which was administered via telephone calls on the 3rd and 7th days after extraction, and the pain, swelling, bleeding, and dry socket parameters were also measured. The Landry, Turnbull, and Howley Index (LWHI) was used to evaluate socket healing after 2 and 4 weeks. The Benex^®^ group reported significantly less pain on the third day. Both groups demonstrated incomplete epithelialization of the sockets in the second week, whereas in the fourth week, 42.1% of the Benex^®^ group showed complete epithelialization, which was significant. Even though the volume changes were not compared, as in our study, it can be assumed that Benex^®^ causes less trauma due to the faster epithelialization of the socket. This method aims to prevent the expansion of the socket walls so that fewer thin adjacent bone walls fracture. We know that, especially in the maxillary anterior region, the buccal bone plate is ≤1 mm and almost 50% of the plates have a thickness of ≤0.5 mm [28]. Therefore, we also assumed that the existing thickness must influence the change in volume due to the extraction method; however, no significant differences in our patient cohort were demonstrated. However, within the study group compared to the control group, we observed that the least volume change occurred at a thickness of less than 1 mm, and the greatest volume change occurred at a thickness greater than 1 mm, even though more initial volume was available in this study. Thus, a thicker >1 mm offers less overall volume stability than a thinner labial cortical plate ≤1 mm, despite the use of Benex^®^, which is a surprising result. In contrast, in the control group, this observation was as expected; specifically, a thicker labial cortical plate >1 mm was more stable in volume. Interestingly, this does not correlate with the thickness of the gingival biotype. Due to the fact that this observation was the same in both groups, a thicker type also showed more volume loss than a thinner type. The analysis of the selected ROIs can serve as an explanation for this effect. The facial ROI with the greatest volume loss was always selected as the reference, as we consider this to be the most relevant at the timepoints t1 and t5. Moreover, the fact that the measurement was performed intraoperatively by the surgeon with a probe might bias this observation.

Various methods, such as clinical, cast model, or radiographic examinations, have already been used in several human studies to evaluate the volume change after extraction [3,28,33,34,35,36,37,38,39]. The method used in this study for the evaluation of volumetric changes over time is established and has been studied in previous research. To minimize the method error, the intraoral scanner was calibrated in accordance with the manufacturer’s instructions, a scan strategy for obtaining digital scans [40], a precise best-fit algorithm for superimpositions of the STL models [41,42], and a well-documented CAD evaluation software for measurement performance that reduces the risk of manual errors [43,44,45,46] were utilized. However, it should be noted that the method error was not separately assessed in this study and can arise during the workflow of the applied techniques, such as deviations in the precision of the intraoral scan, selection of the valid surface area, or changes in the evaluation software.

Due to the fact that the soft tissue follows the hard tissue in the remodeling processes, we used intraoral scanning as the methodology. Based on the remodeling processes that have already been demonstrated in animal studies [28,47,48], we chose the corresponding time points with the maximum timepoint of 90 days, as bone modeling appears to occur earlier than remodeling in the first 3 months, and the greatest shape changes would be expected in this time period [3]. Based on our own preliminary research [43,49] as well as other research [23,50], intraoral scanning is considered a proven method of assessing the soft tissue or volume and contour changes of the alveolar process or socket. Particularly with regard to the socket, retrospective research using intraoral scanning was able to show a significant loss of volume due to the similar methodology and overlaying of the stl. files as in our study [23]. The only difference is that we divided the sockets into somewhat dedicated ROIs and assessed the effects of the neighboring teeth on the change in volume after extraction. With the adjacent tooth present, whether mesial or distal, there was less volume loss in the corresponding ROIs at time t5 than in the reference, with the largest loss in ROI3. This difference was also significant but not in the comparison of the control and study groups. However, a tendency toward the Benex^®^ system could again be observed in this study, which may be due to the small group size.

Several other limitations (other than the sample size and the absence of method error assessment) should be mentioned and discussed. The thickness of the soft tissue and bone was only determined intraoperatively by the surgeon tactile by a probe; thus, biases would be present. In the presence of an adjacent tooth mesial and distal, there was less volume loss, and the thickness of the gingiva or the labial cortical plate had no significant influence on the volume of the facial aspect of the socket at different time points, but the determination by a probe might not be sufficiently predictable.

CBCT images provide better correlations with IO scans and evaluations. Obviously, this must always be performed with the goal of justifying X-ray indication, which was not considered in this study. In addition, PROMs should be collected when using such a technique to determine their possible clinical relevance. However, in this study, volume changes after different extraction methods could be observed through the evaluation of different IO scans.

## 5. Conclusions

Due to the small patient cohort, the results are limited and not sufficiently interpretable. However, the greatest volume loss was observed in the facial aspect of the socket regardless of the utilized extraction method, although this loss tended to be less with Benex^®^.

## Figures and Tables

**Table 1:** Definition of the dimensions of the teeth as the basis for the definition of the ROI cuboid in the specific ROI.

Tooth	Length	Width	Height
Anterior teeth	25 mm	16 mm	20 mm
Canine	30 mm	16 mm	20 mm

**Table 2:** Explanation of the parameters used to determine the volumetric change between the different and matched ROIs.

Parameter	Importance
Valid surface area (Area)	Surface area in mm^2^
Total volume (Vol)	Total volume (integrated distance) in mm^3^
Quotient total volume/valid surface area (Vol/Area)	Ratio of total volume to surface area in mm^3^/mm^2^

**Table 3:** Detailed information on the included patients according to the control and study groups and the parameters of sex, age, tooth extraction, localization of the teeth, and nicotine abuse status are shown.

Parameters	Numbers in n=	Groups
Sex	Male n = 13	Control: n = 7, Study: n = 6
	Female n = 17	Control: n = 9, Study: n = 8
Age (Average and ±SD)	65.4 ± 12.0	Control: 65.9 ± 13.1
		Study: 64.9 ± 11.0
Teeth	Total: n = 43	Control: n = 23, Study: n = 20
Localization of teeth	Tooth: 11/21, n = 14	Control: n = 9, Study: n = 5
	Tooth: 12/22, n = 17	Control: n = 8, Study: n = 9
	Tooth: 13/23, n = 12	Control: n = 6, Study: n = 6
Nicotine abuse	Nonsmokers, n = 19	Control: n = 9, Study: n = 10
	≤10 cig./day, n = 5	Control: n = 3, Study: n = 2
	>10 cig./day, n = 6	Control: n = 4, Study: n = 2

**Table 4:** Detailed values within the control group for the change in volume of the ratio between total volume and area in mm^3^/mm^2^ of the ROI_3, ROI_4, ROI_5, ALLmesial, and ALLdistal areas at the timepoints t1 and t5. The table contains the calculated arithmetic mean values (Mean) with the corresponding standard deviations (SDs), as well as the largest and smallest (Min) values. The table contains the calculated arithmetic mean values (Mean) with the corresponding standard deviations (SD) as well as the largest (Max) and smallest (Min) values.

ROI	Timepoint	Mean	SD	Min	Max
ROI_3	t1	−0.101	0.682	−1.058	1.659
	t5	−1.598	0.657	−3.252	−0.417
ROI_4	t1	0.258	0.799	−1.283	2.161
	t5	−1.007	0.681	−2.165	0.345
ROI_5	t1	0.291	0.693	−1.081	1.723
	t5	−0.703	0.643	−1.767	0.821
ALLmesial	t1	0.110	0.434	−0.711	1.029
	t5	−0.603	0.377	−1.269	0.086
ALLdistal	t1	0.034	0.417	−0.616	1.071
	t5	−0.690	0.480	−1.880	0.298

**Table 5:** Detailed values within the study group for the change in volume of the ratio between total volume and area in mm^3^/mm^2^ of the ROI_3, ROI_4, ROI_5, ALLmesial, and ALLdistal areas at t = 1 and t = 5. The diagram contains the calculated arithmetic mean values (Mean) with the corresponding standard deviations (SDs), as well as the largest and smallest (Min) values. The table contains the calculated arithmetic mean values (Mean) with the corresponding standard deviations (SD) as well as the largest (Max) and smallest (Min) values.

ROI	Timepoint	Mean	SD	Min	Max
ROI_3	t1	−0.193	0.441	−1.147	0.657
	t5	−1.396	0.605	−2.763	−0.685
ROI_4	t1	0.307	0.484	−0.302	1.384
	t5	−0.587	0.468	−1.328	0.929
ROI_5	t1	0.192	0.353	−0.323	0.990
	t5	−0.615	0.356	−1.135	0.079
ALLmesial	t1	0.014	0.145	−0.384	0.276
	t5	−0.406	0.289	−1.094	0.079
ALLdistal	t1	−0.060	0.178	−0.338	0.410
	t5	−0.645	0.417	−1.479	−0.104

**Table 6:** Comparison of the volume change per area (mm^3^/mm^2^) between the ROIs (ROIs_3, 4, 5, ALLmesial, and ALLdistal) at timepoint t5 and among the groups (control vs. study group). The intercept value indicates the mean value of the volume change predicted by the model for the reference group. At this point, this is equal to ROI_3 of the control group at time t1. * Significance level *p* < 0.05.

Variable	Coefficient	95% CI (Lower)	95% CI (Upper)	*p* Value
Intercept	−0.393	−0.555	−0.231	<0.001 *
ROI_4	0.559	0.423	0.694	<0.001 *
ROI_5	0.615	0.480	0.751	<0.001 *
ROI_ALLdistal	0.485	0.349	0.620	<0.001 *
ROI_ALLmesial	0.601	0.466	0.736	<0.001 *
timepoint t5	−0.919	−1.005	−0.834	<0.001 *
Comparison of groups	0.062	−0.131	0.254	0.533

**Table 7:** Comparison of the volume change per area (mm^3^/mm^2^) between the ROIs (ROI_1, 3 and ALLmesial) at timepoint t5 and considering the mesial adjacent tooth ATmesial (present vs. not present) and the other groups (control vs. study group). * Significance level *p* < 0.05.

Variable	Coefficient	95% CI (Upper)	95% CI (Lower)	*p* Value
Intercept	−1.884	−2.121	−1.646	<0.001 *
ATmesial	0.442	0.195	0.688	0.001 *
ROI_1	0.592	0.414	0.770	<0.001 *
ROI_ALLmesial	0.993	0.814	1.171	<0.001 *
Comparison of the groups	0.242	0.001	0.484	0.060

**Table 8:** Comparison of the volume change per area (mm^3^/mm^2^) between the ROIs (ROI_3, 7 and ALLdistal) at timepoint t5 and taking into account the mesial adjacent tooth ATdistal (present vs. not present) and the other groups (control vs. study group). * Significance level *p* < 0.05.

Variable	Coefficient	95% CI (Upper)	95% CI (Lower)	*p* Value
Intercept	−1.747	−2.015	−1.479	<0.001 *
ATdistal	0.300	0.032	0.568	0.037 *
ROI_7	0.374	0.192	0.557	<0.001 *
ROI_ALLdistal	0.835	0.652	1.018	<0.001 *
Comparison of the groups	0.192	−0.076	0.461	0.174

## Data Availability

The datasets used and/or analyzed during the current study are available from the corresponding author upon reasonable request.
